# Two-in-one procedure for transvenous lead extraction and leadless pacemaker reimplantation in pacemaker-dependent patients with device infection: streamlined patient flow

**DOI:** 10.1093/europace/euae162

**Published:** 2024-07-20

**Authors:** Baptiste Maille, Nathalie Behar, Peggy Jacon, Jerome Hourdain, Frederic Franceschi, Linda Koutbi, Lilith Tovmassian, Cédric Bierme, Elena Seder, Victor Klein, Martin Postzich, Noemie Resseguier, Christophe Leclerq, Pascal Defaye, Jean-Claude Deharo

**Affiliations:** Department of Cardiology, Timone University Hospital, 264 rue Saint-Pierre, 13005 Marseille, France; Department of Cardiology and Vascular Disease Division, Rennes University Hospital, Rennes, France; Department of Cardiology, CHU Grenoble Alpes, Grenoble, France; Department of Cardiology, Timone University Hospital, 264 rue Saint-Pierre, 13005 Marseille, France; Department of Cardiology, Timone University Hospital, 264 rue Saint-Pierre, 13005 Marseille, France; Department of Cardiology, Timone University Hospital, 264 rue Saint-Pierre, 13005 Marseille, France; Department of Cardiology, Timone University Hospital, 264 rue Saint-Pierre, 13005 Marseille, France; Department of Cardiology, Timone University Hospital, 264 rue Saint-Pierre, 13005 Marseille, France; Department of Cardiology, Timone University Hospital, 264 rue Saint-Pierre, 13005 Marseille, France; Department of Cardiology, Timone University Hospital, 264 rue Saint-Pierre, 13005 Marseille, France; CEReSS-Health Service Research and Quality of Life Center, School of Medicine, Aix-Marseille University, Marseille, France; CEReSS-Health Service Research and Quality of Life Center, School of Medicine, Aix-Marseille University, Marseille, France; Department of Cardiology and Vascular Disease Division, Rennes University Hospital, Rennes, France; Department of Cardiology, CHU Grenoble Alpes, Grenoble, France; Department of Cardiology, Timone University Hospital, 264 rue Saint-Pierre, 13005 Marseille, France

**Keywords:** Leadless pacemaker, Transvenous lead removal, Device-related infection

In pacemaker-dependent (PMD) patients, treatment of device-related infection (DRI) leads to prolonged hospital stays^1,2^ and exposes patients to specific complications of temporary pacing, including lead dislodgement, cardiac tamponade, and reinfection of the temporary lead.^3^ Prolonged hospitalization also independently increases the risk of adverse events and morbidities.^4,5^ Therefore, strategies that can lead to simpler patient flow and shorter hospital stays are needed.^6^

Limited data suggest that leadless pacemaker (LPM) implantation can be safely performed in the context of DRI.^7–10^ The objective of our multi-centre study was to prospectively assess whether a two-in-one procedure of transvenous lead removal (TLR) followed by immediate reimplantation with an LPM was feasible and safe and could shorten hospital stays of PMD patients with DRI.

We prospectively enrolled all PMD patients who underwent successful TLR of an infected device followed by immediate reimplantation of an LPM during a single procedure at three French academic centres between January 2018 and December 2022. Patients, at the time of TLR, with active bacteraemia and/or uncontrolled clinical sepsis and patients with a clear indication for resynchronization therapy were excluded.

Standardized follow-up was performed with the aim of detecting any signs of DRI relapse, any reason for reintervention on the reimplanted system, or the occurrence of complications. We collected the proportions of patients alive with a cured DRI at 1 month after the procedure and the mid-term survival rate. The cure of DRI was considered as control of sepsis and the absence of DRI recurrence.^11^ The complication survival rate was evaluated during the entire follow-up and included those related to TLR, DRI, antibiotic therapy, hospital stay, reimplantation, and any other cardiovascular adverse events. In order to evaluate patient flow, the numbers of days alive and out of hospital and the number of days in the intensive care unit (ICU) until the 30th day after the TLR were calculated. Outcomes were then compared with a historical cohort (*n* = 30) of all consecutive patients who had received TLR and delayed reimplantation, as recommended, from January 2014 to January 2018 at one of the three centres.^1^ The study was approved and recorded by our institutional ethic committee with CSE23-45 number.

A total of 45 PMD patients had a two-in-one extraction/reimplantation procedure (*Figure 1*), after a median interval time of 4 (1–9) days after tertiary hospital admission. They all had negative blood cultures at the time of the procedure, the last positive blood cultures before TLR having been obtained at a median [interquartile range (IQR)] of 15(8–19) days. Median (IQR) follow-up was 186 (86–365) days. One foreign patient was lost to follow-up after being alive at hospital discharge on Day 11 and was excluded from the outcome analyses. Two patients experienced sepsis worsening despite the two-in-one procedure and died. The first was a 99-year-old man who died because of the ongoing sepsis the day after the procedure. The other was an 84-year-old woman, being the only patient among the 22 (4.5%) device-related endocarditis patients, who had a recurrent methicillin-resistant *Staphylococcus aureus* positive blood cultures 5 days after the two-in-one procedure. However, neither repeated transthoracic echography nor computed tomography could identify any sign of LPM infection or secondary localization of endocarditis. She died 13 days after procedure. One month after the two-in-one procedure, 41/44 patients (93.2%) were alive with a cured DRI.

**Figure 1:**
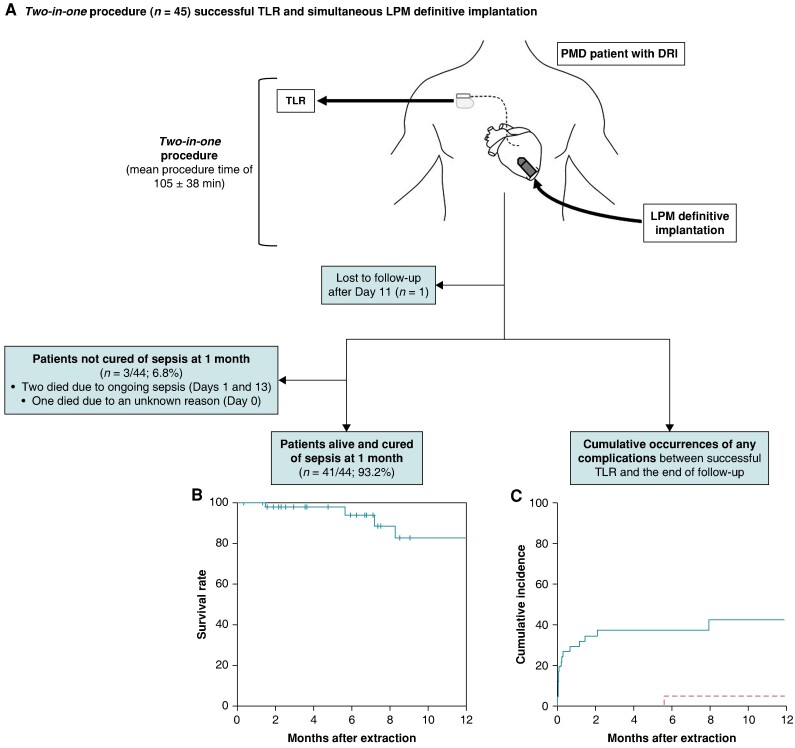
Two in one procedure of TLR and immediate reimplantation with an LPM in patients with cardiac electronic device infection. (*A*) The primary approach consisted of TLR. During the extraction procedure, pacing was performed using a temporary wire placed within the right ventricle via femoral access. Immediately after the extraction procedure, an LPM (Micra AV or Micra VR, Medtronic) was implanted through a new femoral 27 Fr sheath, within the right ventricle. (*B*) Long-term survival rate in patients alive and cured of sepsis at 1 month. (*C*) Cumulative incidence of complication after TLR. Complications included those related to TLR, DRI, antibiotic therapy, hospital stay, reimplantation (i.e. the reimplantation procedure, and the reimplanted device follow-up), and any other cardiovascular adverse events. Between successful TLR and the end of follow-up, solid line illustrated the cumulative occurrences of any complications, taking into account the risk of death (dotted line) as a competing event. DRI, device-related infection; LPM, leadless pacemaker; PMD, pacemaker dependent; TLR, transvenous lead removal.

At the end of follow-up, 9/44 patients (20.5%) had died. Among patients alive and free of sepsis at 1 month, mid-term overall survival is illustrated in *Figure 1*. This was not significantly different between the two cohorts (crude hazard ratio = 1.11; 95% confidence interval = 0.25–5.04; *P* = 0.88). The complication-free survival rate of patients with a cured DRI at 1 month was 27/44 (61.4%). Between successful TLR and the end of follow-up, the cumulative occurrence of any complications is illustrated in *Figure 1*. Detailed patient outcomes during the first month and over the whole follow-up period are described in Supplementary material online, *Tables S1* and *S2*. No evidence of LPM dislodgment was reported. However, an 86-year-old man died suddenly the night following the two-in-one procedure. The cause of death remained unknown but occurred while the patient was sleeping in a conventional ward without electrocardiogram monitoring. No autopsy was performed. The mean (standard deviation) number of days alive and out of hospital during the 30 days after the TLR was significantly longer in the single procedure cohort vs. the historical cohort (*Table 1*). This reduction in the length of hospital stay appears to be partly related to a reduction in ICU stay.

**Table 1:** Baseline characteristics

	Two-in-one procedure cohort	Historical cohort	*P*
Age (years)	79.6 (10.4)	79.1 (11.3)	0.84
Male	34 (75.6%)	21 (70.0%)	0.59
Underlying type of cardiac pathology			0.81
None or hypertensive	19 (42.2%)	14 (46.7%)	
Ischaemic, valvular, dilated, or congenital	26 (57.8%)	16 (53.3%)	
LVEF, %	54.5 (8.6)	47.9 (13.0)	0.01
Diabetes mellitus	11 (24.4%)	5 (16.7%)	0.42
Serum creatinine, µmol/L	99.0 (78.0–129.0)	106.5 (81.8–120.0)	0.29
Infected CIED			0.58
Pacemaker without resynchronization	37 (82.2%)	22 (73.3%)	
Implantable cardioverter-defibrillator	2 (4.4%)	1 (3.3%)	
Cardiac resynchronization	6 (13.3%)	7 (23.3%)	0.09
Number of leads per patient,			
1	3 (6.7%)	1 (3.3%)	
2	31 (68.9%)	15 (50.0%)	
3	10 (22.2%)	9 (30.0%)	
≥4	1 (2.2%)	5 (16.7%)	
Age of the oldest lead, years	10.6 (6.3)	10.8 (8.5)	0.93
Type of infection			0.01
Pocket infection only	23 (51.1%)	25 (83.3%)	
Device-related endocarditis	22 (48.9%)	5 (16.7%)	
Vegetation status prior TLR			
Lead vegetation	15 (33.3%)	4 (13.3%)	0.05
Valve related vegetation	2(4.4%)	1 (3.3%)	0.81
Valve related vegetation after TLR	2(4.4%)	2(6.7%)	0.67
Bacterial culture identification			
Gram-positive bacteria:	34 (75.6%)	20 (66.7%)	
*S. aureus*	8	10	
*CN staphylococci*	15	9	
*Strepto/enterococcus* spp.	7	0	
*Propionibacterium* spp.	6	3	
Gram-negative bacteria	4 (11.1%)	10 (33.3%)	
*Fungi*	0	1	
Culture negative	6 (13.3%)	2	
Antibiotics duration, days	16 (10–40)	21 (9–42)	0.76
In case of pocket infection	10 (2–15)	21 (7–28)	
In case of endocarditis	40 (28–42)	42 (28–63)	
Type of reimplanted device:			
Immediate LPM reimplantation	45 (100%)		
Micra VR	35 (77.8%)		
Mivra AV	10 (22.2%)		
Delayed reimplantation		29 (97.7%)	
Single-chamber device		10 (33.3%)	
Dual-chamber device		11 (36.7%)	
Resynchronization therapy		8 (26.7%)	
ICD		6 (20%)	
Epicardial reimplantation		5 (16.7%)	
Number of days alive and out of hospital during the 30 days after the TLR, days	22.2 (8.6)	17.6 (7.3)	0.02
Duration of stay before TLR, days	8.0 (10.6)	4.9 (3.0)	0.13
ICU duration stay, days	1.2 (2.7)	7.0 (4.9)	<0.001
Patients alive at 1 month after TLR with a cured DRI	41 (93.2%)	26(86.7%)	0.35
Complication-free survival rate of patients with a cured DRI at 1 month	27(61.4%)	18 (60.0%)	0.91

Data are *n* (%), mean (SD), or median (IQR).

CIED, cardiac implantable electronic device; ICU, intensive care unit; IQR, interquartile range; LPM, leadless pacemaker; LVEF, left ventricular ejection fraction; SD, standard deviation; CN, coagulase negative; TLR, transvenous lead removal; DRI, device-related infection.

Our study is the first to include prospective and comparative data regarding the potential benefit on patients’ flow of two-in-one procedure. Recently, Beccarino *et al*.^7^ have reported for the first time the retrospective assessment of a cohort of DRI patients who had immediate reimplantation of an LPM after TLR. Their results are similar to our prospective cohort in terms of feasibility and safety. Interestingly, even though they enrolled a significant proportion of patients with active bacteraemia (38% of whom had positive blood cultures within 72 h of the procedure) and implanted the LPM immediately before TLR, only 6% of their patients had persistent positive blood cultures after TLR, and they did not report recurrence of DRI with the same organism during follow-up. However, they observed one extraction failure (which was successful in a second procedure) and short lead remnants were left in place in three patients. In contrast, we opted for temporary pacing during TLR and LPM implantation if TLR was successful and uneventful. We believe that both approaches have their own advantages and disadvantages, but our sequential approach may prove to be more reasonable in the event of TLR failure, which may occur in 3.3% of the cases,^12^ avoiding implantation of an LPM in a patient with infected leads still in place. Of note, the temporary wire was not associated with any complications in our cohort.

One-month mortality in our cohort (6.8%) and long-term high mortality rate (*Figure 1*) likely reflects the age and comorbidities of the patients included in TLR studies, as previously described,^8,13–15^

Given the limited number of patients enrolled, attributable to the relatively low occurrence of DRI and PMD cases, it is imperative to interpret our findings cautiously and validate them through dedicated randomized trials. In the meantime, however, our study confirms the feasibility and safety of such an approach. It also complements previous studies by demonstrating more efficient patient pathways, with implications for economic considerations and the incidence of in-hospital complications.^4,5^

## Supplementary Material

euae162_Supplementary_Data

## Data Availability

The data underlying this article will be shared on reasonable request to the corresponding author.
