# Supplementary material for: Two-in-one procedure for transvenous lead extraction and leadless pacemaker reimplantation in pacemaker-dependent patients with device infection: streamlined patient flow
Source: Europace. 2024 Jul 20;26(7):euae162. doi: 10.1093/europace/euae162 (PMC11259849; doi:10.1093/europace/euae162)
Supplement: euae162_Supplementary_Data [file euae162_supplementary_data.docx]

**Supplemental Table 1** Type and timing of outcomes in the Two-in-one cohort (*n* = 44)

| **Type of event** | **First month** | **Beyond 1 month (mid-term follow-up)** |
| --- | --- | --- |
| DRI not cured | - 2 patients died of septic shock 1 and 13 days after the Two-in-one procedure |  |
| Complications related to hospital stay | - 2 urinary tract infections | - 1 death secondary to aspiration pneumonia |
| Complications and deaths related to initial DRI or extraction | - 6 acute renal insufficiencies - 2 extraction pocket hematomas - 1 sudden unexplained death in the 24 hours after the extraction procedure - 1 serious cutaneous allergic reaction secondary to antibiotic treatment - 1 pancytopenia | - 3 heart failure symptoms due to tricuspid regurgitation |
| Complications related to CIED reimplantation | - 1 early ventricular pacing threshold elevation | - 1 pacemaker syndrome |
| Complications related to any other cardiovascular adverse event | - 1 heart failure symptoms (due to ventricular asynchronism and severe mitral regurgitation) | - 2 deaths due to heart failure (both patients had severe LVEF dysfunction after CIED extraction) |
| Non-cardiac deaths |  | - 3 non-cardiac deaths |

CIED, cardiac implantable electronic device; DRI, device-related infection; LPM, leadless pacemaker; LVEF, left ventricular ejection fraction.

**Supplemental table 2** Type and timing of outcomes in the historical cohort (*n* = 30)

| **Type of event** | **First month** | **Beyond 1 month (mid-term follow-up)** |
| --- | --- | --- |
| DRI not cured | - 2 deaths due to worsening of sepsis (days 2 and 12) - 1 vegetation on temporary lead |  |
| Complications related to hospital stay | - 1 aspiration pneumonia - 1 urinary tract infection - 1 death due to in-hospital COVID-19 infection - 1 failure to thrive |  |
| Complications related to initial DRI or extraction | - 1 acute renal insufficiency - 1 pancytopenia | - 1 heart failure symptoms secondary to severe tricuspid regurgitation |
| Complications related to CIED reimplantation | - 2 cardiac tamponades |  |
| Complications related to any other cardiovascular adverse event |  |  |
| Non-cardiac deaths |  | - 7 non-cardiac deaths |

CIED, cardiac implantable electronic device; DRI, device-related infection; COVID-19, Coronavirus disease 2019.
